# An unusual cause of difficulty in walking downstairs: Focal task‐specific dystonia in the lower limb

**DOI:** 10.1002/ccr3.2894

**Published:** 2020-05-13

**Authors:** Takafumi Hasegawa

**Affiliations:** ^1^ Division of Neurology Department of Neuroscience & Sensory Organs Tohoku University Graduate School of Medicine Sendai Japan

**Keywords:** downstairs, dystonia, kicking movement, lower limb, task‐specific

## Abstract

Stair‐specific FTSD in the lower limb is very rare. Most patients show dystonic posture when walking downstairs rather than upstairs.

## Graphical abstract

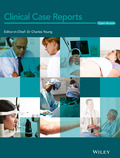

Dystonia is a neurological disorder that causes involuntary, excessive muscle contraction. We report a 59‐year‐old woman with focal task‐specific dystonia (FTSD) when going downstairs.

In her early 20s, she exhibited a weird movement on her leg when descending stairs. Her symptom was characterized by a patterned “kicking movement” only when walking downstairs (Video [Link], [Link]). Neurological examination and brain MRI showed no abnormality. Stair‐specific FTSD is a rare clinical entity. Most patients show dystonic posture when walking downstairs, and two phenotypes (kicking and lifting type) have been described.^1^, ^2^ Pharmacological intervention usually fails, but the symptoms remain unchanged over a long time.

## CONFLICT OF INTEREST

None declared.

## AUTHOR CONTRIBUTIONS

TH: involved in the acquisition of data, supervised the study, analyzed and interpreted the data, and wrote the paper.

## Supporting information

Video S1

Sup info
