# Supplementary material for: An unusual cause of difficulty in walking downstairs: Focal task‐specific dystonia in the lower limb
Source: Clin Case Rep. 2020 May 13;8(8):1595. doi: 10.1002/ccr3.2894 (PMC7455451; doi:10.1002/ccr3.2894)
Supplement: Supplementary file 2 — Sup info [file CCR3-8-1595-s002.docx]

Video legend

The patient shows abnormal dystonic posture in her right leg when descending the stairs. Going up stairs is essentially normal.
